# The effect of biosimilar administration on clinical outcomes in patients with adalimumab‐controlled psoriasis

**DOI:** 10.1002/ski2.60

**Published:** 2021-09-18

**Authors:** M. Panahi, Y. Skelly, R. Zaman

**Affiliations:** ^1^ Department of Dermatology Hull University Teaching Hospitals NHS Trust, Castle Hill Hospital Cottingham UK

## Abstract

**Background:**

Adalimumab is an anti‐tumour necrosis factor administered for the management of severe psoriasis. Previously limited to Humira, new biosimilar medications have now emerged including Amgevita. To date, there have been no comparison studies of adalimumab biosimilar use on different types of psoriasis.

**Objective:**

To investigate the implications of biosimilar medications and patient specific factors on clinical outcomes, including Psoriasis Area and Severity Index (PASI) and Dermatology Life Quality Index (DLQI) scores.

**Methods:**

A clinical notes review was performed for all dermatology patients with adalimumab‐controlled psoriasis at our centre. Demographic profile, psoriasis subtype and changes in clinical patterns as demonstrated by PASI and DLQI were extracted and analysed.

**Results:**

Of 91 records identified, 70 patients met the inclusion criteria. 21 patients (30%) demonstrated significant increase in PASI and DLQI scores with Amgevita. Scores improved to baseline once Humira was restarted. Findings reveal no difference in pre‐adalimumab disease severity or mean age between the groups. Patients responding only to Humira had a greater proportion of females, and were likelier to have psoriatic arthritis (odds ratio [OR]: 10.63; *p* < 0.0002) and nail involvement (OR: 6.13, *p* < 0.02), compared with patients well controlled with Amgevita.

**Conclusions:**

This audit of a single dermatology centre suggests switching to a biosimilar adalimumab may exacerbate symptoms of psoriasis. Future studies should investigate whether findings are restricted to our study population, and consider the influence of other factors, such as disease subtypes and medication formulations.

## Graphical abstract

Clinical outcomes of patients with adalimumab‐controlled psoriasis administered Humira and its biosimilar Amgevita were compared. Patients responding only to Humira had a greater proportion of females, and were likelier to have psoriatic arthritis (odds ratio (OR): 10.63; *p* < 0.0002) and nail involvement (OR: 6.13, *p* < 0.02), compared with patients well controlled with Amgevita. This audit supports our re‐trialling of biosimilar drugs prior to switching biological class in patients with psoriatic arthritis.

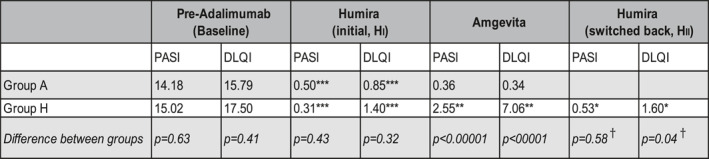

1

**What is already known about this topic?**

- Adalimumab is an anti‐tumour necrosis factor medication licenced for the treatment of moderate‐to‐severe psoriasis which has not been controlled by standard systemic non‐biological therapies.
- Amgevita is a bio‐similar of Humira.
- Both drugs have demonstrated similar outcomes in patients with plaque psoriasis and rheumatoid arthritis.

**What does this study add?**

- Once biosimilar have been shown to be of comparable quality, extrapolation of indications occur, allowing for the approval of a new biosimilar for all the licenced indications of the referenced drug, in the absence of approved clinical data for each study.
- To date, there have been no comparison studies of adalimumab biosimilar use in different types of psoriasis, in particular, patients with psoriatic arthritis.
- Our independent dermatology department conducted a clinical audit, which has identified an inconsistency in symptom control with the different medications, supporting the need for further research into adalimumab and its biosimilars.

## 2 INTRODUCTION

Psoriasis is a chronic inflammatory skin condition characterised by the abnormal proliferation and differentiation of keratinocytes.^1^ It is estimated to affect 2% of the UK population,^2^ ranging from mild disease to an extensive debilitating condition with substantial physical and psychosocial consequences.^1^

Adalimumab is a recombinant human monoclonal antibody that inhibits the pro‐inflammatory cytokine tumour necrosis factor alpha (TNF‐α).^3^, ^4^ It has been licenced for the management of moderate to severe psoriasis as determined by a Psoriasis Area Severity Index (PASI) score of 10 or more, a Dermatology Life Quality Index (DLQI) over 10, or for patients who have not responded to standard systemic non‐biological therapies.^3^

Initially adalimumab was limited to Humira, however, biosimilar medications have recently been introduced including Amgevita.^5^ Biosimilars are highly similar versions of the biological with comparable pharmacological activity, quality and safety.^6^

Following the trust approval of Amgevita in 2018, all patients with adalimumab‐controlled psoriasis under the care of our dermatology department were switched from Humira to Amgevita. During routine 3‐monthly biological clinic appointments, some patients previously well‐controlled with adalimumab began reporting symptom exacerbation with increased PASI and DLQI scores. Each patient case was individually assessed by a senior clinician to rule out external factors such as acute illness, injury or seasonal fluctuations of symptoms, before offering patients the option to switch back to Humira.

Some patients reported an improvement of symptoms once Humira was restarted. Data was required to quantify patients' switch back to Humira and identify whether symptoms were significant enough to support the decision of restarting Humira. There was a need to identify which factors influence the differences in clinical outcomes between both medications. We examined potential associations between medication and disease severity, demographics and subgroups of psoriasis.

## 3 METHODS

### 3.1 Ethics approval

The audit was approved by the Hull and East Yorkshire Hospitals NHS Trust Clinical Audit and Effectiveness Team (CG1: 2020.037). Patient consent was not obtained, as only anonymous data was extracted. This was stored on a secure database with access restricted to the authors.

### 3.2 Derivation of audit data

A retrospective clinical note review was performed. We obtained data from clinical notes, patient questionnaire scores and electronic health records. Data collected included age, sex, psoriasis type and disease severity measured at baseline and during 12‐weekly routine clinic appointments. For patients who were administered long‐term Humira prior to the introduction of Amgevita, mean questionnaire scores from the previous 5 years of follow‐up were used. PASI and DLQI scores were considered outliers in circumstances where a change was attributable to a known stressor, such recent surgery, and these were removed from the final database.

### 3.3 Participants

All adult patients with psoriasis who were previously administered Humira for at least 6 months, and automatically switched to the bioequivalent drug Amgevita between 1 January 2018 and 1 November 2019 were included in the audit. Patients were identified using pharmacy records and dermatology clinical notes.

Exclusion criteria included recent drug holidays, patients lost to follow‐up or mortality, and patients residing outside the trust, where notes were not delivered during the auditing period.

### 3.4 Statistical analysis

Data analysis was performed with SPSS software (SPSS Version 26; SPSS inc) to determine statistical significance. Chi squared test was performed to compare gender. Student *T*‐test was performed to compare age and differences between PASI and DLQI scores at specific time‐intervals; *p* values were two‐tailed, and *p* values less than 0.05 were considered statistically significant. Data were expressed as mean plus or minus standard deviation unless otherwise indicated. The comparison of outcomes based on subtype of psoriasis was analysed using the odds ratio (OR).

## 4 RESULTS

During the audit data collection period (1 April 2020 to 30 May 2020) a total of 91 patients were identified, of which 70 met the inclusion criteria. Of these, 21 patients (30%) were switched back to Humira. Reasons noted for restarting Humira consisted of: flare of plaques (6 patients, 28.6%), joint pain (4 patients, 19.1%) or both (11 patients, 52.4%). Alongside symptom exacerbation, one incident of flushing and weight gain was documented.

The patient demographics and clinical characteristics are summarised in Table 1. Of note, the mean age of participants continuing to be administered Amgevita, *group A*, was 56 (range 47–87, median 55, interquartile range 46–65), and 53 (range 33–60, median 54, interquartile range 51–56) for Humira, group H, with no difference between the groups (*p* < 0.04). Patients switched back had a greater proportion of females compared with patients continuing Amgevita (9 (47.6%) and 10 (21.4%), respectively; *p* < 0.03). There was no significant difference in baseline disease severity prior to commencing adalimumab.

**TABLE 1:** Baseline demographics of patients in the audit

	Patients continuting Amgevita Group A (*N* = 49)	Patient swiched back to Humira Group H (*N* = 21)
Age, median years (IQR)	56 (47–87)	53 (30–60)
Female gender	10 (21%)	9 (48%)
Baseline PASI, median score (IQR)	14 (10–18)	15 (12–17)
Baseline DLQI, median score (IQR)	16 (10–21)	18 (13–21)
Treatment		
Humira (initial)^*^, median months (IQR)	83 (63–122)	69 (42–89)
Amgevita, median months (IQR)	‐	6 (3–8)
Previous biological therapy		
Etanercept	11 (22%)	4 (19%)
Usteskinumb	1 (2%)	1 (5%)
Comorbidities		
Obesity	8 (16%)	4 (19%)
Hypertension	9 (18%)	3 (14%)
Type 2 diabetes	4 (8%)	2 (10%)
Degenerative/muscular disease	4 (8%)	3 (14%)
Psychological	3 (6%)	2 (10%)
Atrial fibrillation	3 (6%)	1 (5%)
Renal failure	3 (6%)	‐
Thyropathy	2 (4%)	‐
Asthma	2 (4%)	‐
Gout	2 (4%)	‐
Myocardial infarction	1 (2%)	‐
Multiple allergies	1 (2%)	‐
Sarcoidosis	1 (2%)	‐
Haemochromatosis	‐	1 (5%)

*Note:* Data are N (%) unless otherwise indicated.

Abbreviations: DLQI, Dermatology Life Quality Index; IQR, interquartile range; PASI, Psoriasis Area and Severity Index.

^*^ Duration of Humira administration, in months, prior to introduction of new biosimilar medication.

Table 2 shows the trend of PASI and DLQI scores. Mean scores have been described except for Amgevita use in group H, where final scores that determined the swap back to Humira (H_II_) are provided. Commencement of adalimumab for the first time significantly improved scores (*p* < 0.00001), with comparable outcomes in both groups. The average duration of treatment with Humira initially (H_I_) was similar in both groups (*p* = 0.65).

**TABLE 2:** Change in PASI and DLQI scores by medication administered in patients with symptoms controlled with Amgevita, group A, compared with patients with symptom control limited to Humira, group H

	Pre‐adalimumab (Baseline)		Humira (initial, H_I_)		Amgevita		Humira (switched back, H_II_)	
	PASI	DLQI	PASI	DLQI	PASI	DLQI	PASI	DLQI
Group A	14.18	15.79	0.50***	0.85***	0.36***	0.34		
Group H	15.02	17.50	0.31***	1.40***	2.55**	7.06**	0.53*	1.60*
Difference between groups	*P* = 0.63	*P* = 0.41	*P* = 0.43	*P* = 0.32	*P* < 0.00001	*P* < 00001	*P* = 0.58^a^	*P* = 0.04^a^

*Note:* Asterisks (*) indicate significant changes in score compared with the prior drug regimen administered in those patients.

Abbreviations: DLQI, Dermatology Life Quality Index; IQR, interquartile range; PASI, Psoriasis Area and Severity Index.

^a^ Difference between the final questionnaire scores of patients remaining on Amgevita and patients switched back to Humira.

*p < 0.005, **p < 0.0005, ***p < 0.00001, unpaired, two‐tailed Student *T*‐Test.

Once Amgevita was started, group A continued to have consistent PASI (*p* = 0.5) and DLQI (*p* = 0.1) scores, whereas group H reported significantly increased average scores (*p* < 0.0005). The average time of Amgevita administration in group H was 27 weeks (range 9–40, median 28, interquartile range 14–35). When the 21 patients in group H with worsening outcomes restarted Humira (H_II_), the PASI and DLQI scores were comparable to the pre‐Amgevita (H_I_) scores (*p* = 0.39 and *p* = 0.88, respectively).

**FIGURE 1:**
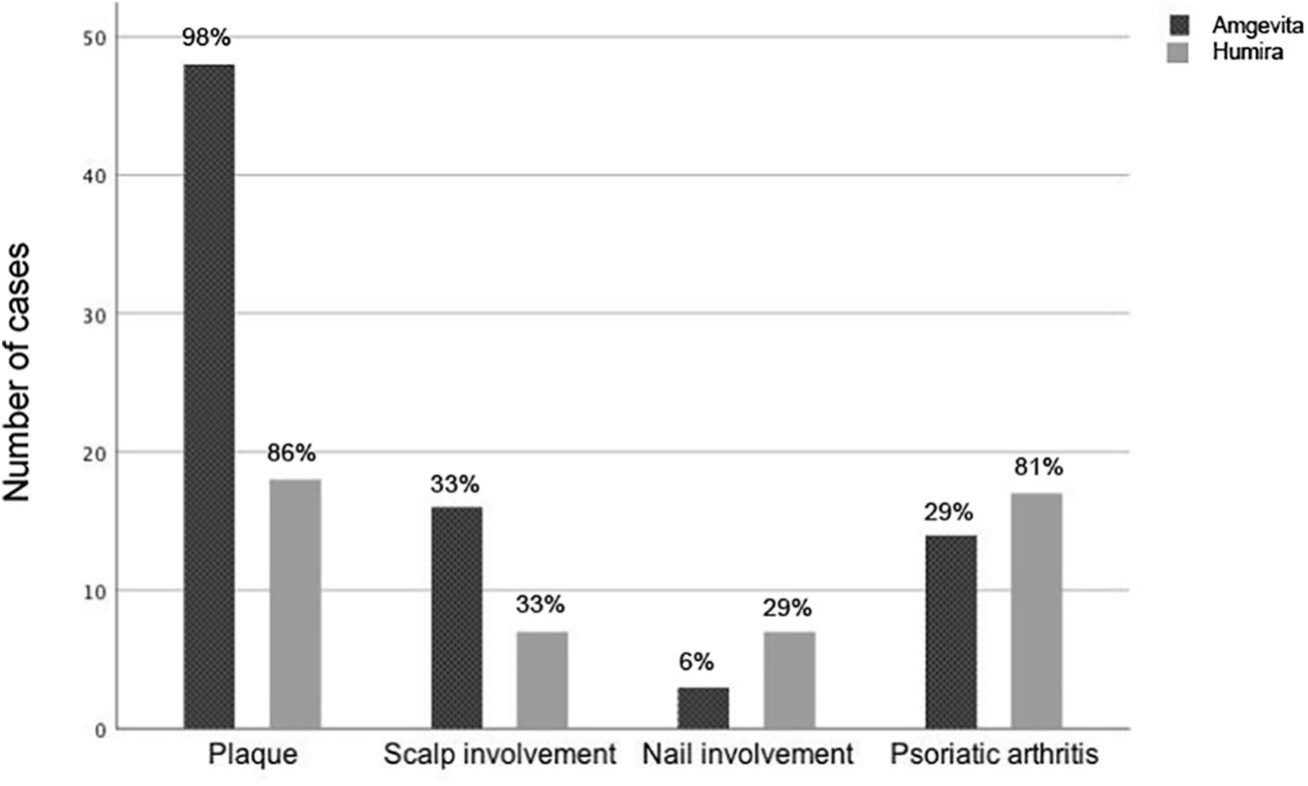
Incidence of psoriasis subtypes in patients with symptom control with Amgevita compared with patients with symptom control limited to Humira. Bars represent number of cases (percentage)

The number of cases for different subtypes of psoriasis in patients taking Amgevita and patients switched back to Humira alongside their prevalence (%) in each group are detailed in Figure 1. Patients could fall into one or multiple subtype categories.

**FIGURE 2:**
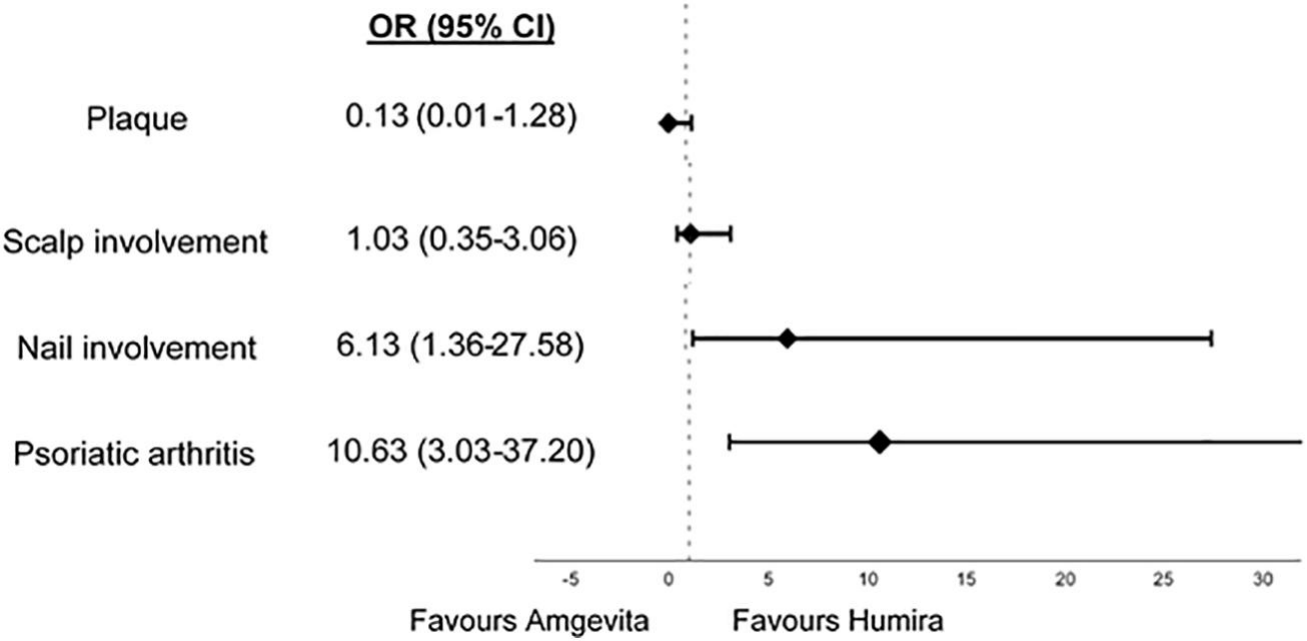
Odds ratio of symptom control with Amgevita compared with Humira in different psoriasis subgroups

The likelihood of disease control with both adalimumab drugs depending on the subtype of psoriasis are presented in Figure 2 with OR and 95% confidence intervals for these patients.

## 5 DISCUSSION

The purpose of our audit was to identify trends in patients who required switching back to Humira, following a pattern of increasing PASI and DLQI scores detected in biological clinics. From this, we aimed to objectively evaluate patient outcomes. Overall, 70% of dermatology patients with psoriasis continued to be administered the trust‐approved adalimumab. The improvement of assessment scores when Humira was restarted in the remaining 30% of the cohort suggests the re‐trialling of a biosimilar could provide better clinical outcomes.

Most patients who required switching back to Humira did so at the 2nd or 3rd 12‐weekly follow‐up clinic after starting Amgevita. More frequent follow‐ups in prospective studies could prove useful in clarifying when this loss of response to a new biosimilar is seen. Patient biases may have influenced results. When initially introduced as the new trust‐approved adalimumab, patients were provided with information detailing the evidence in support of Amgevita and its possible side effects profile. Many of these patients had used Humira for a long time with excellent symptom control. A new treatment may have therefore been met with patient bias through lack of compliance or confidence in the new medication.^7^

Importantly, these findings reflect the outcomes of 91 patients in a single dermatology outpatient centre and larger population studies are required to determine their significance. The retrospective nature of audits limits the data available to that which has already been documented. Whilst patients are screened for comorbidities, health conditions that arise after commencing biologicals and conditions managed solely by primary care who use different computer systems could be missing from the secondary care notes. Despite accessing both electronic and written records, only 9 of the 91 patients had their ethnic group (White, British) recorded. Given the genetic component of psoriasis,^2^ comparing patient ethnicities with their response to biosimilars may be of value.

Both plaque psoriasis and psoriatic arthritis have complex, multifactorial aetiology with both genetic and environmental influence.^1^, ^8^ Although gender incidence of psoriasis appears to be equal, differences in the severity and distribution of psoriasis remain poorly understood.^9^, ^10^ Our audit identified a significantly higher percentage of females in the group of patients who required switching back. Interestingly, some studies have identified a pattern of earlier discontinuation of anti‐TNF agents in females with psoriatic arthritis, compared to males,^11^, ^12^ attributed to a combination of higher likelihood of reduced response to treatment and adverse drug effects.^11^ The audit indicates female patients continued to exhibit improved symptom control once Humira was restarted. To account for gender differences in psoriasis presentation and response to biologicals, future studies should compare the underlying pathophysiology in males and females.

Guidelines recommend waiting 16 weeks for clinical response to Adalimumab before considering alternative drugs.^3^ This is not required with biosimilars; in previous studies, single transition from Humira to Amgevita has been demonstrated not to influence drug benefits or adverse effects.^13^, ^14^ Nonetheless, the majority of patients who required switching back did so following this waiting period, reducing the likelihood of acute illness or flares affecting patient outcomes.

Survival bias may also have affected the patients who were included in the audit. Patients who develop response failure to both drugs are switched to another class of biological drug and these patients were consequently excluded during data collection.

Secondary failure is a common phenomenon when administering biotherapies. Biological drugs elicit an immune response, forming anti‐drug antibodies, often against the antigen binding site, neutralising its effects or causing unwanted reactions.^6^, ^15^ Development of binding and neutralising antibodies have been shown to be similar in Amgevita and Humira comparison studies.^16^, ^17^ Patients who responded poorly to Amgevita demonstrated improved PASI and DLQI scores once Humira was restarted when compared to previous Humira use, reducing the likelihood of the difference in scores during Amgevita being attributed to immunogenicity. However, the DLQI questionnaire scores in group A, continuing Amgevita had a small but significant (*p* = 0.04) advantage over group H, which could be attributed to secondary failure.

Amgevita was licenced following extensive in vitro and phase I studies to ensure safety and quality,^5^ and its long‐term efficacy and effects were studied in phase III equivalence studies. Both drugs were shown to have similar outcomes in patients with plaque psoriasis^13^, ^16^ and rheumatoid arthritis.^17^ Once shown to be of comparable quality, extrapolation of indications may occur, allowing for the approval of a new biosimilar for all the licenced indications of the referenced drug, in the absence of approved clinical data for each study.^6^ Therefore, studies comparing the exchange of Humira for Amgevita in patients with psoriatic arthritis are not available for comparison with our findings. In addition to the active drug component, injections include additives including buffers to stabilise the formula. Variations in these additives may also influence clinical outcomes and require further examination.

## 6 CONCLUSION

The expiration of patents for biological medications allows new drugs to be introduced into the pharmaceutical market. This increases manufacturing competition, reduces cost and therefore improves accessibility of these medications.

The majority of patients with psoriasis successfully switched to the new biosimilar, maintaining symptom‐control. However, in a subset of patients, increasing PASI and DLQI scores suggest that switching to a biosimilar could exacerbate psoriasis. A future prospective study with a large sample size is required to confirm the significance of our findings. As biologicals and biosimilars become increasingly used, the results of such a study will allow physicians to make better decisions for their patients.

## CONFLICT OF INTEREST

None to declare.

## AUTHOR CONTRIBUTIONS

**M. Panahi:** Data curation; Formal analysis; Investigation; Methodology; Project administration; Software; Visualization; Writing – original draft; Writing – review & editing. **Y. Skelly:** Conceptualization; Data curation; Project administration; Resources; Software; Writing – review & editing. **R. Zaman:** Conceptualization; Data curation; Funding acquisition; Resources; Supervision; Validation; Writing – review & editing.

## Data Availability

The data are not publicly available due to privacy or ethical restrictions.
